# Supplementary material for: MAP4 kinase-regulated reduced CLSTN1 expression in medulloblastoma is associated with increased invasiveness
Source: Sci Rep. 2025 Jan 6;15:946. doi: 10.1038/s41598-024-84753-x (PMC11704044; doi:10.1038/s41598-024-84753-x)
Supplement: Supplementary file 8 — Supplementary Material 8 [file 41598_2024_84753_MOESM8_ESM.pdf]

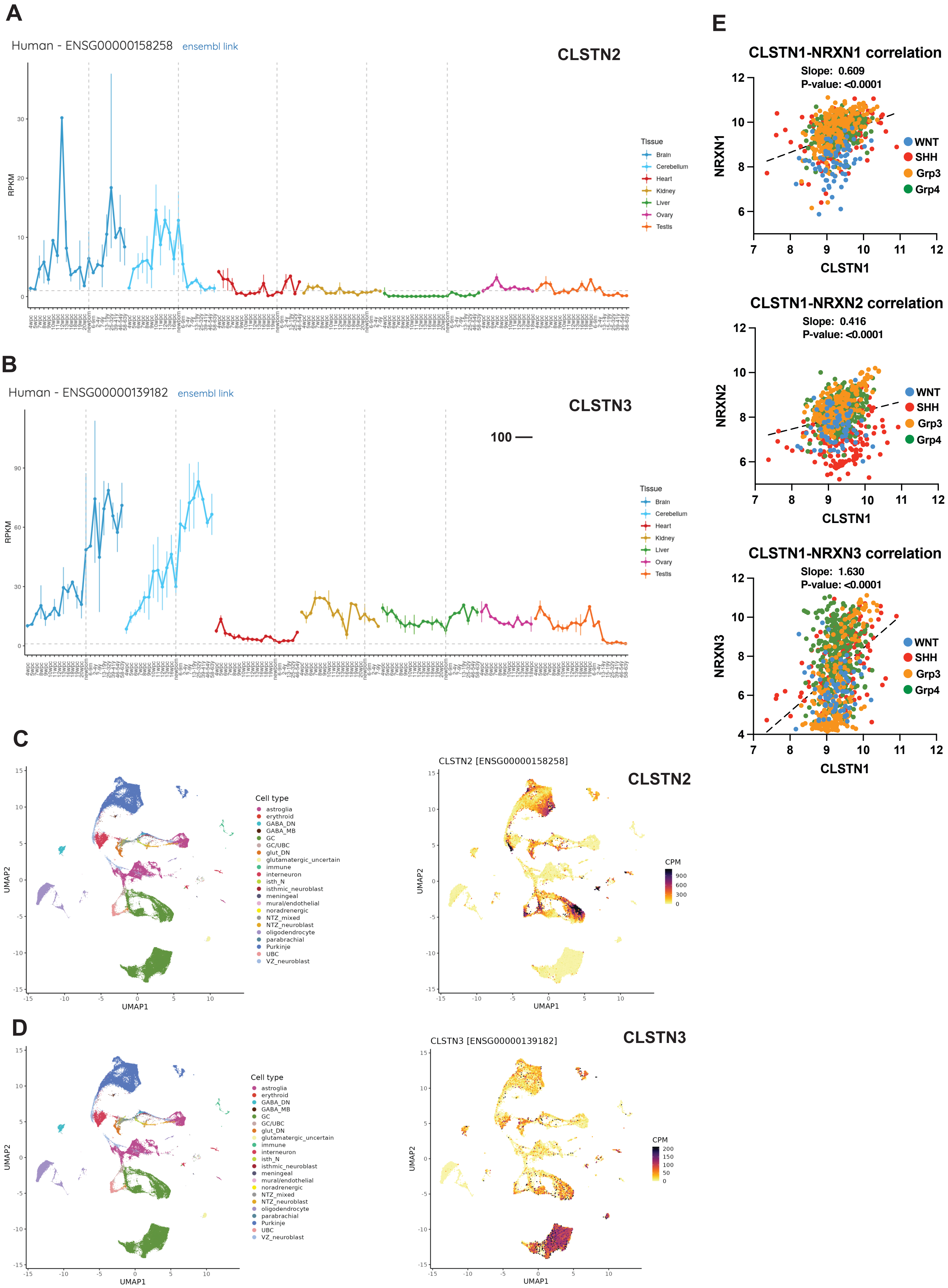

Figure S1:

CLSTN2 and CLSTN3 are differentially expressed during organ development. Analysis of CLSTN2 **(A)** and CLSTN3 **B)** expression across human organ development. snRNAseq analysis of CLSTN2 **(C)** and CLSTN3 **(D)** expression across cerebellar cell types expression. Left: Manifold Approximation and Projection (UMAP) of 180,956 human cells colored by cell type (from48). Right: Expression levels of CLSTN1 across projected cell types. CPM, Counts per million; GABA, Gamma-aminobutyric acid, DN, Dentate nucleus; GC, Granule cells; UBC, Unipolar brush cells; Glut_DN, Glutamatergic deep nuclei neurons; Isth_N, Isthmic nuclei neurons; NTZ, Nuclear transitoryzone; VZ, Ventricular zone. **E)** Correlation of CLSTN1 expression with NRXN1-3 across MB subgroups.

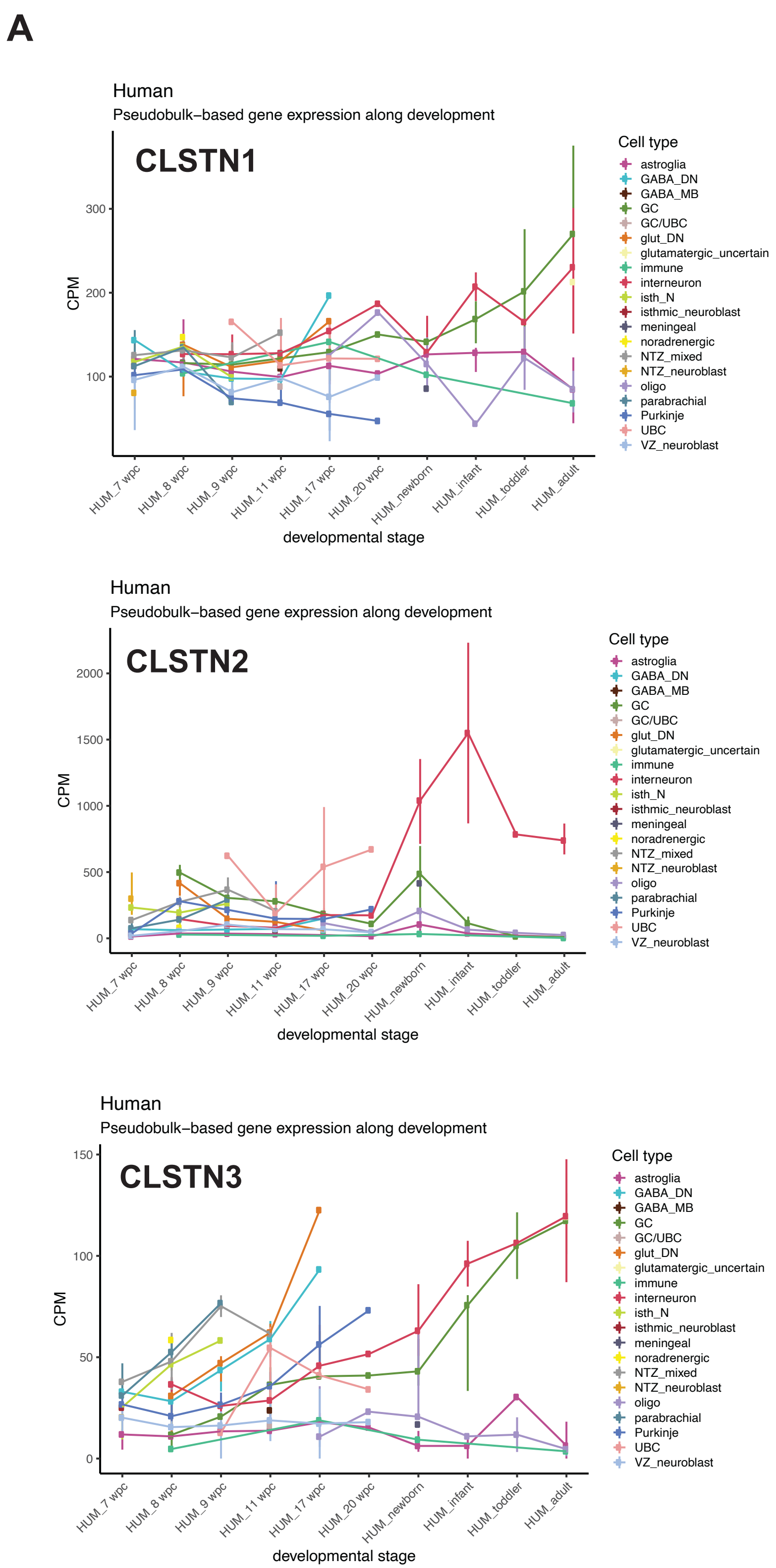

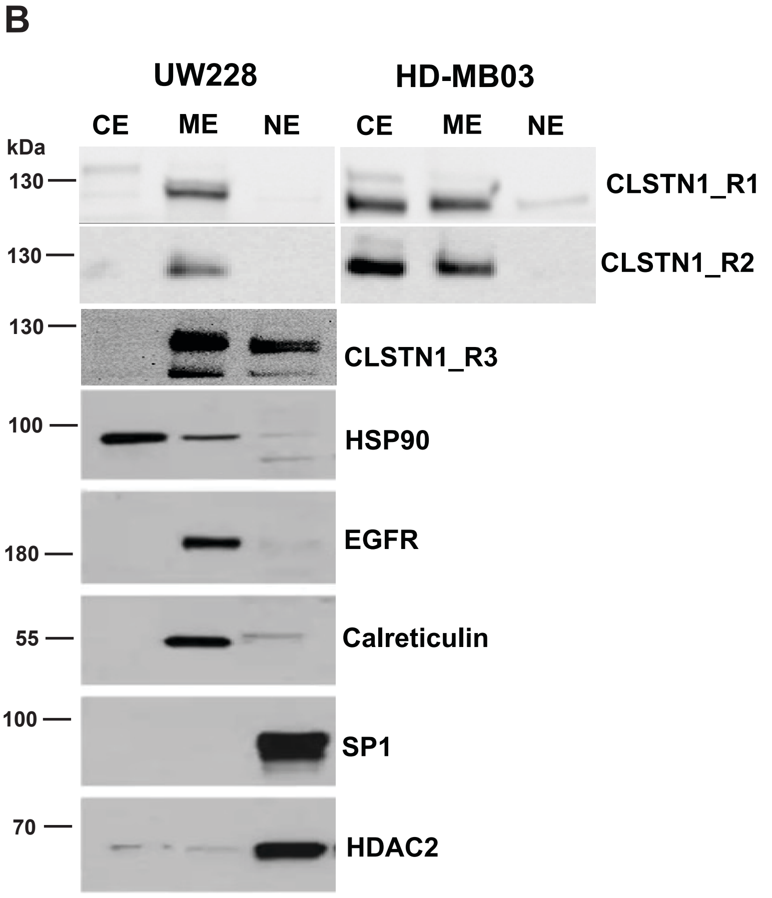

Figure S2

A) Pseudobulk gene expression analysis of CLSTN1, CLSTN2 and CLSTN3 along cerebellar development. **B)** Three (UW228) and two (HD-MB03) IB analyses of CLSTN1 subcellular distribution in cytosolic (CE), membrane (ME) and nuclear (NE) fractions.

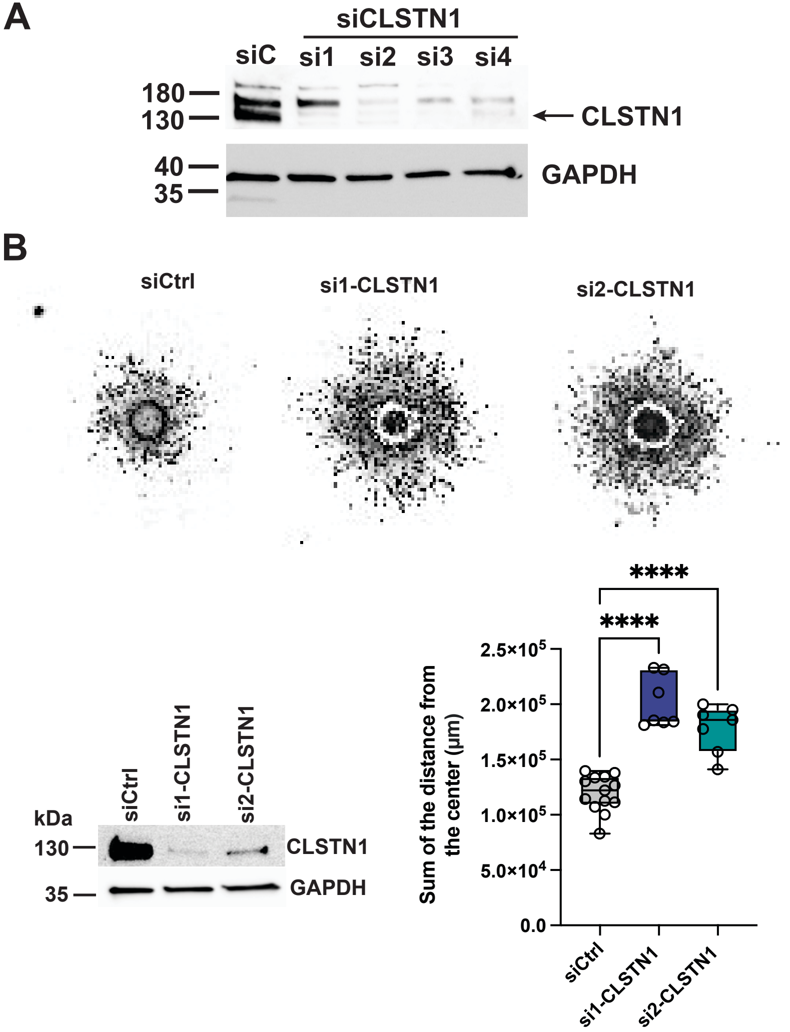

Figure S3:

A) Comparison of depletion efficacy of four different siRNAs targeting CLSTN1.
B) si1-CLSTN1 and si2-CLSTN1 are comparably efficient in increasing bFGF-induced invasion of the tumor cells. Upper panel: SIA of DAOY cells stimulated with 100 ng/ml bFGF for 24 h. Representative images of Hoechst-stained cells at endpoint are shown. Lower left panel depicts the IB validation of siRNA efficacy 48h after transfection. Lower right panel box-dot plot depicts median cumulated

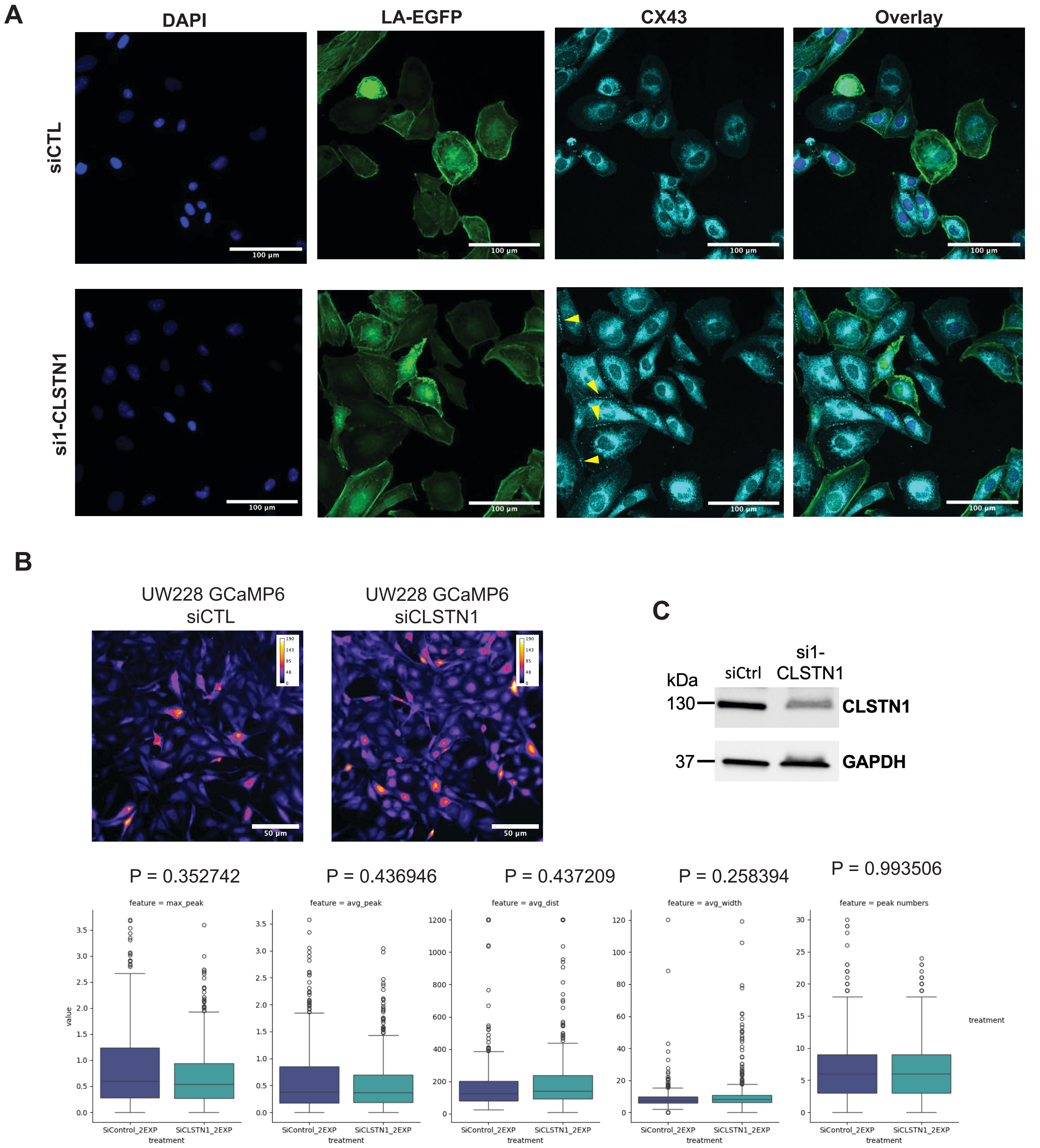

Figure S4:

Depletion of CLSTN1 causes a moderate increase in CX43 in cell-cell contacts without affecting Ca2+ signaling. **A)** IFA analysis of CX43 expression in siCTL or si1_CLSTN1-transfected UW228 cells. Green: LA-EGFP, cyan: CX43. Yellow arrowheads point towards CX43-positive cell-cell contacts. **B)** Quantification of [Ca2+]i using the genetically encoded GCaMP6 calcium sensor in UW228 cells. **C)** IB validation of siRNA-mediated CLSTN1 depletion.

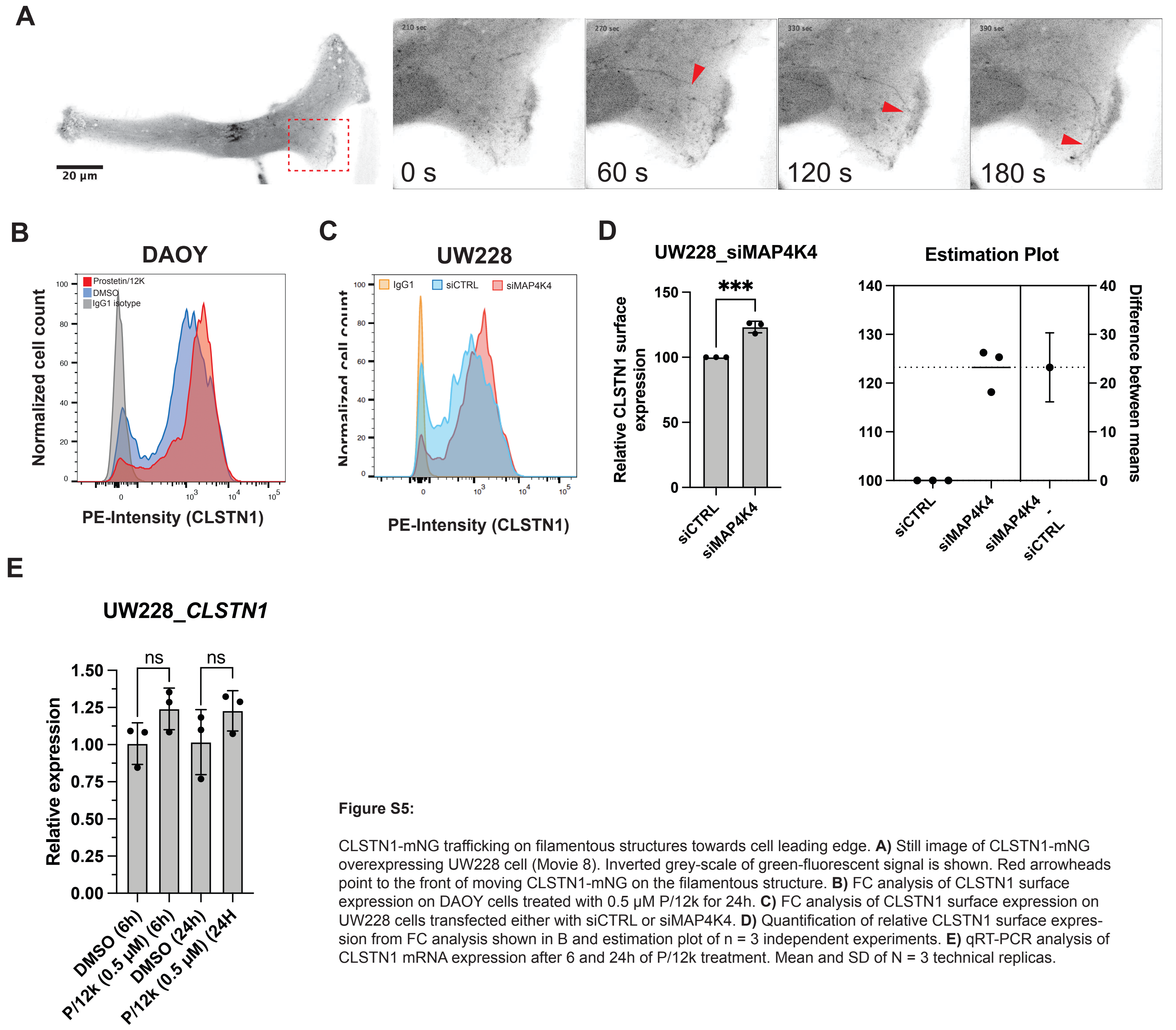

Figure S5:

CLSTN1-mNG trafficking on filamentous structures towards cell leading edge. **A)** Still image of CLSTN1-mNG overexpressing UW228 cell (Movie 8). Inverted grey-scale of green-fluorescent signal is shown. Red arrowheads point to the front of moving CLSTN1-mNG on the filamentous structure. **B)** FC analysis of CLSTN1 surface expression on DAOY cells treated with 0.5 µM P/12k for 24h. **C)** FC analysis of CLSTN1 surface expression on UW228 cells transfected either with siCTRL or siMAP4K4. **D)** Quantification of relative CLSTN1 surface expression from FC analysis shown in B and estimation plot of n = 3 independent experiments. **E)** qRT-PCR analysis of CLSTN1 mRNA expression after 6 and 24h of P/12k treatment. Mean and SD of N = 3 technical replicas.

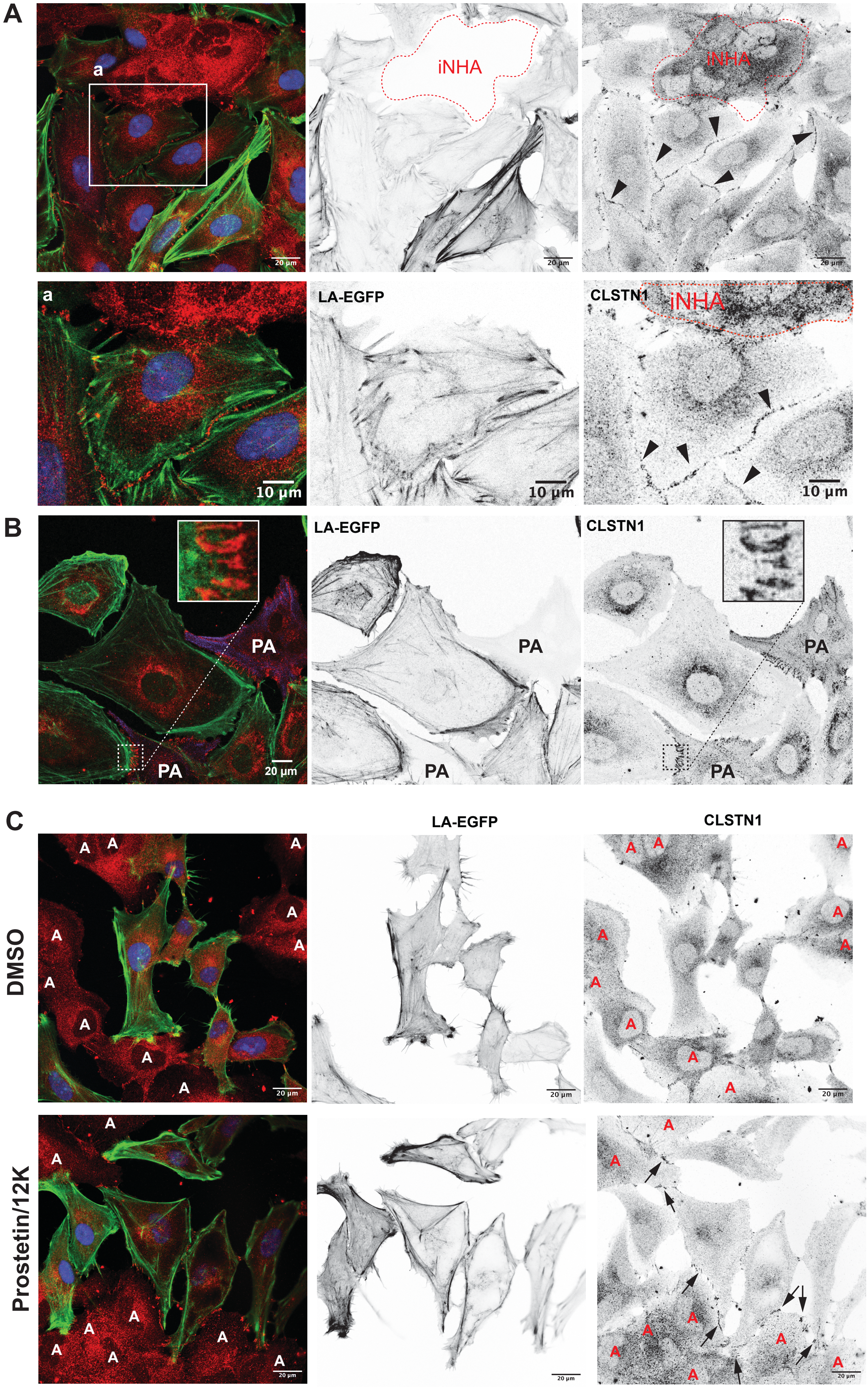

Figure S6:
A) IFA analysis of CLSTN1 expression in UW228-LA-EGFP-mCherry-Nuc cells co-cultured with iNHA. Red: CLSTNA1, green: actin (LA-EGFP), blue: Nuclei (mCherry-Nuc). Letter "A "indicates the positions of iNHAs. Magnification in a) highlights cell-cell contacts between tumor cells. Arrowheads point towards CLSTN1-positive cell-cell contacts between tumor cells. **B)** IFA analysis of CLSTN1 expression in UW228-LA-EGFP-mCherry-Nuc cells co-cultured with primary murine cerebellar astrocytes. Red: CLSTNA1, green: actin (LA-EGFP), blue: Nuclei (mCherry-Nuc), purple: GFAP. Square highlights CLSTN1-positive contact between UW228 tumor cells and astrocytes. **C)** Comparative IFA analysis of CLSTN1 expression in UW228-LA-EGFP-mCherry-Nuc cells co-cultured with iNHA, without or with 0.5 µM P/12k for 24h. Red: CLSTNA1, green: actin (LA-EGFP), blue: Nuclei (mCherry-Nuc). Arrows point towards CLSTN1-positive cell-cell contacts between UW228 and iNHAs. Letter "A "indicates the positions of iNHAs.

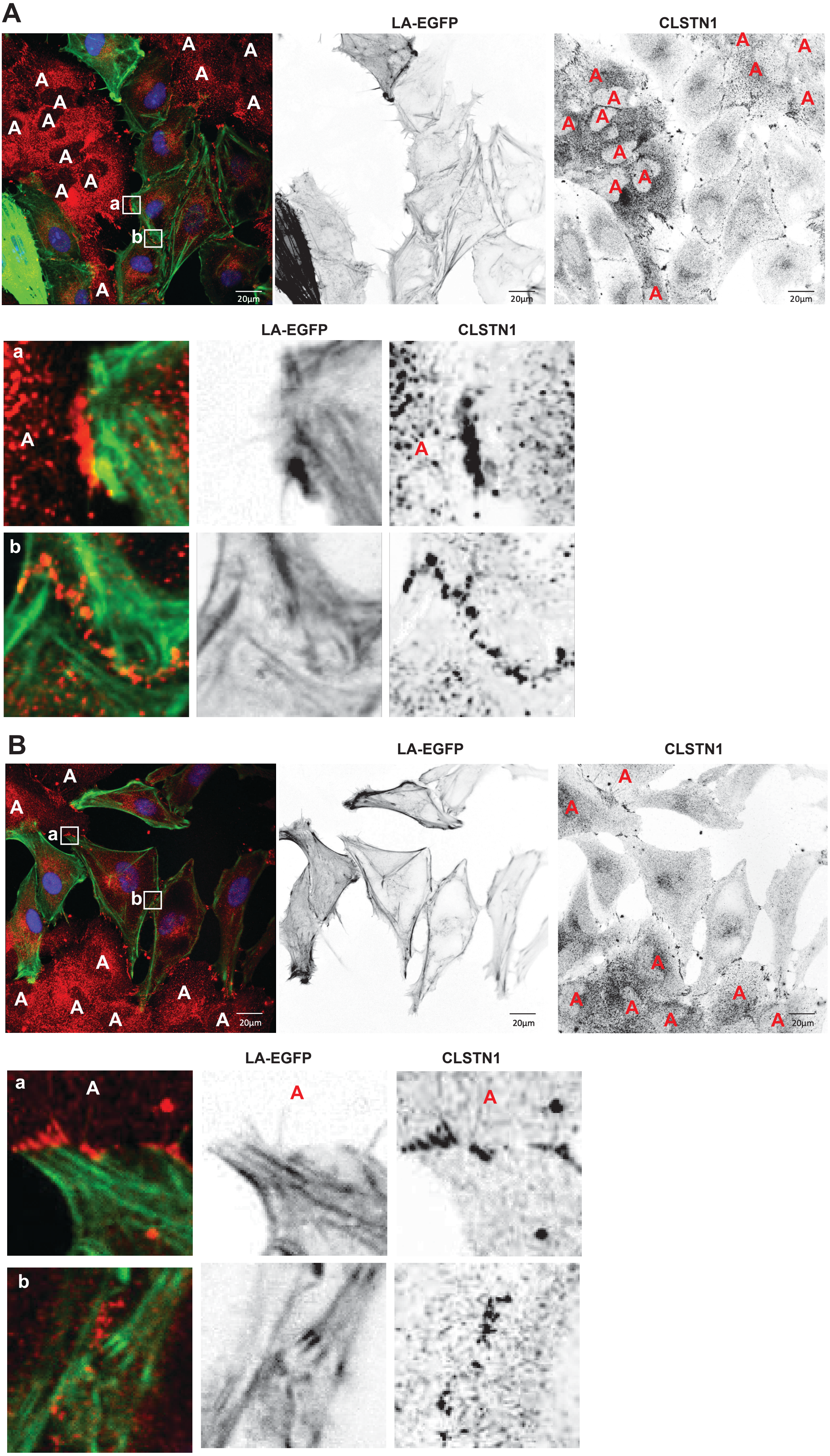

Figure S7:

A) and **B)** show two different samples of UW228-LA-EGFP-mCherry-Nuc cells co-cultured with iNHA in the presence of 0.5 µM P/12k for 24h. Magnifications a) UW228-iNHA CLSTN1-positive cell-cell contacts, b) UW228-UW228 CLSTN1-positive cell-cell contacts. Letter “A “indicates the positions of iNHAs.
