# Supplementary material for: MAP4 kinase-regulated reduced CLSTN1 expression in medulloblastoma is associated with increased invasiveness
Source: Sci Rep. 2025 Jan 6;15:946. doi: 10.1038/s41598-024-84753-x (PMC11704044; doi:10.1038/s41598-024-84753-x)
Supplement: Supplementary file 9 — Supplementary Material 9 [file 41598_2024_84753_MOESM9_ESM.pdf]

Fig. 2A

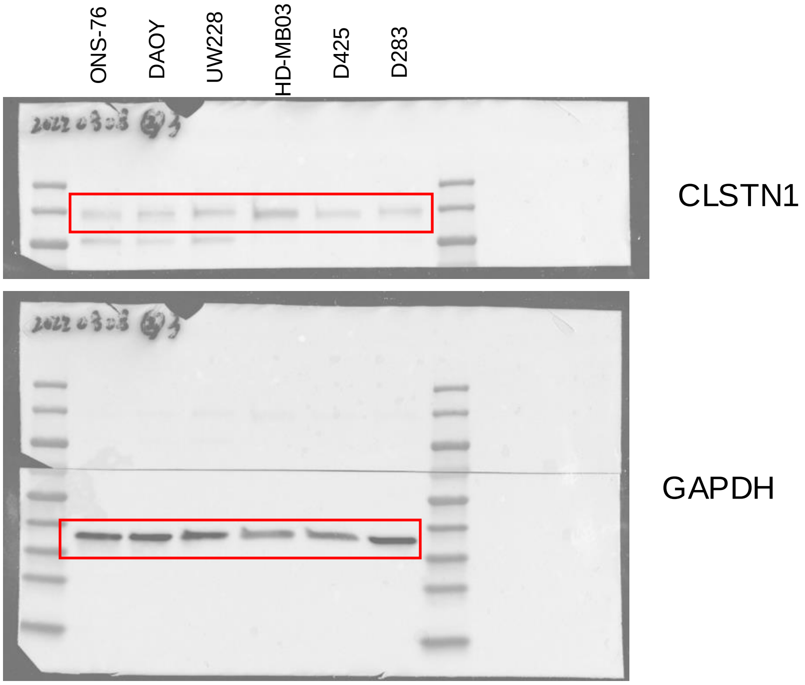
ONS-76
DAOY
UW228
HD-MB03
D425
D283
CLSTN1
GAPDH

Fig. 2C

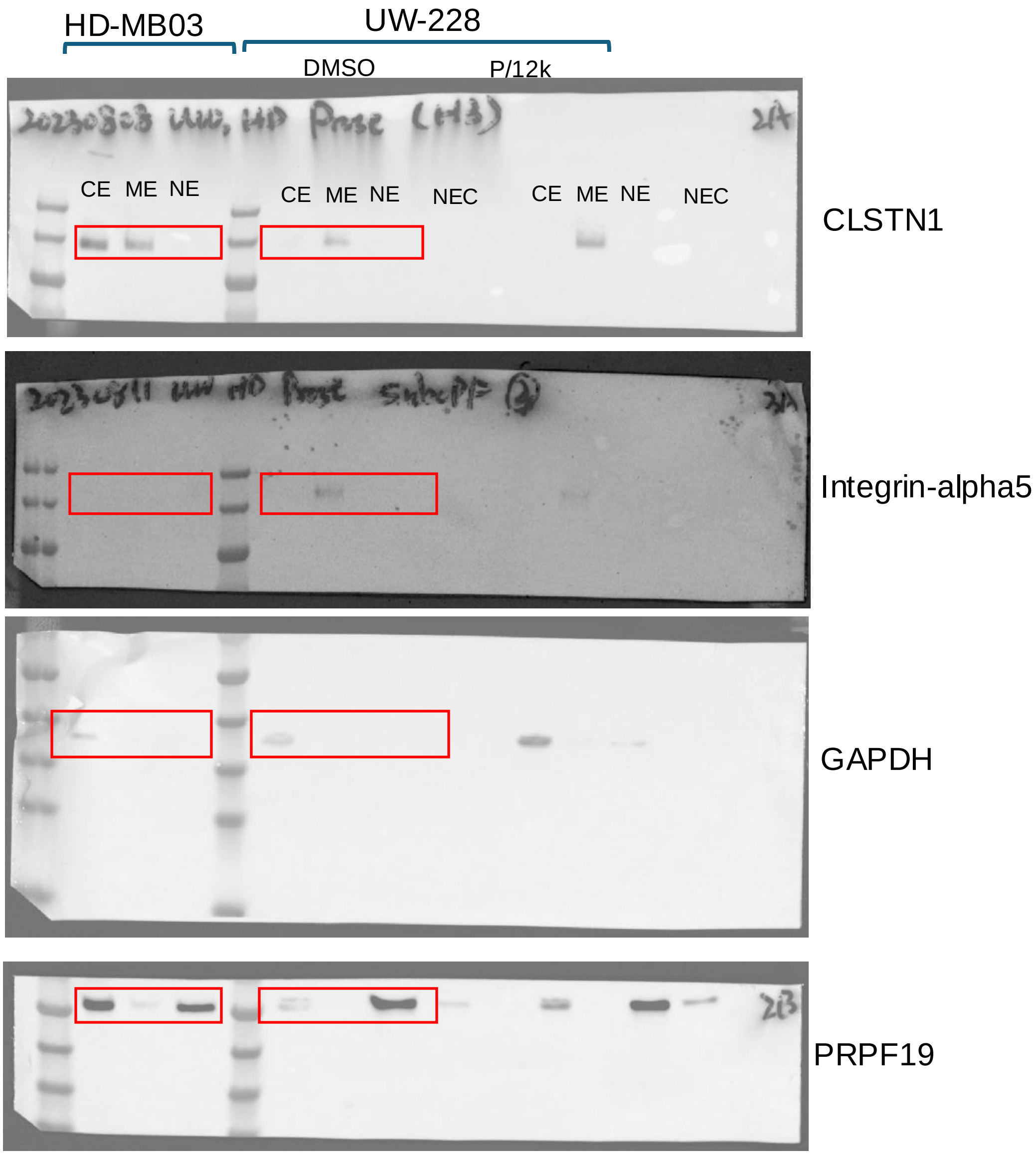
HD-MB03
UW-228
DMSO
P/12k
CE ME NE
CE ME NE
NEC
CE ME NE
NEC
CLSTN1
Integrin-alpha5
GAPDH
PRPF19

Fig. 3A

DAOY

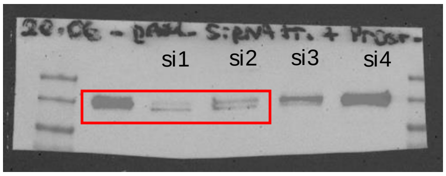

CLSTN1

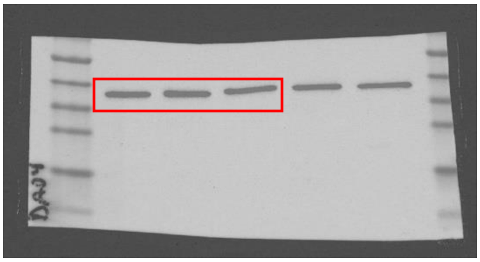

GAPDH

ONS-76

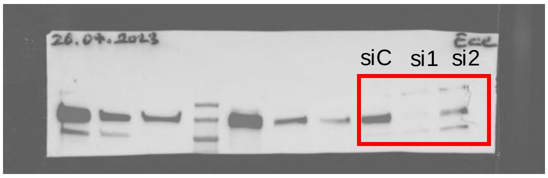

CLSTN1

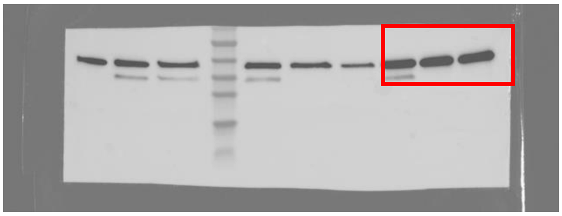

GAPDH

UW228

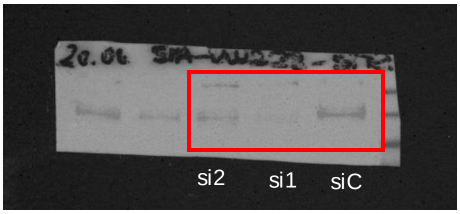

CLSTN1

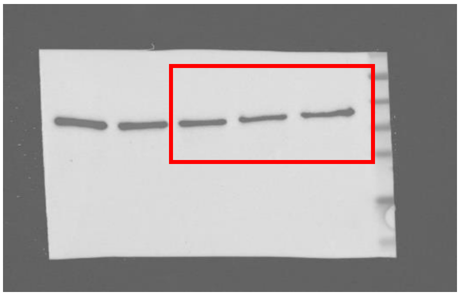

GAPDH

IB is flipped horizontally in Fig. 3A to display it in the same layout as for the other lines

HD-MB03

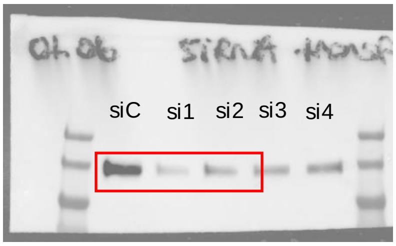

CLSTN1

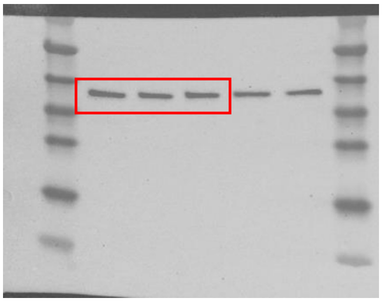

GAPDH

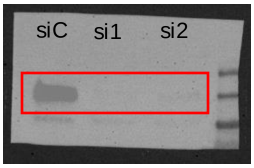

CLSTN1

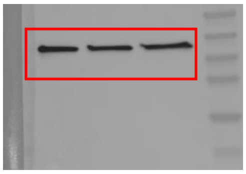

GAPDH

Fig. 3D

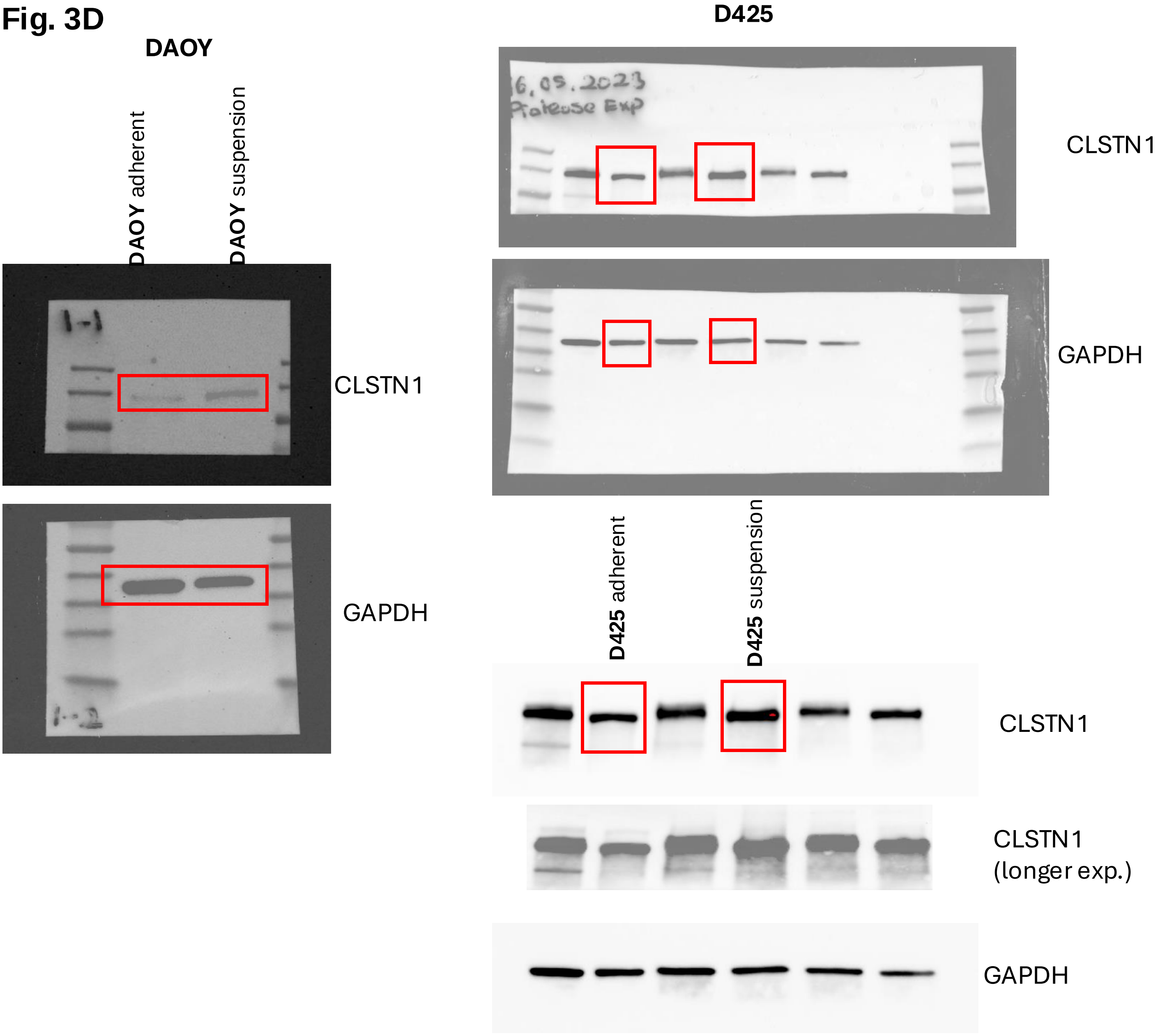

DAOY
D425
DAOY adherent
DAOY suspension
1-1
CLSTN1
GAPDH
1-2
16.05.2023
Protease Exp
CLSTN1
GAPDH
D425 adherent
D425 suspension
CLSTN1
CLSTN1
(longer exp.)
GAPDH

Fig. 3E

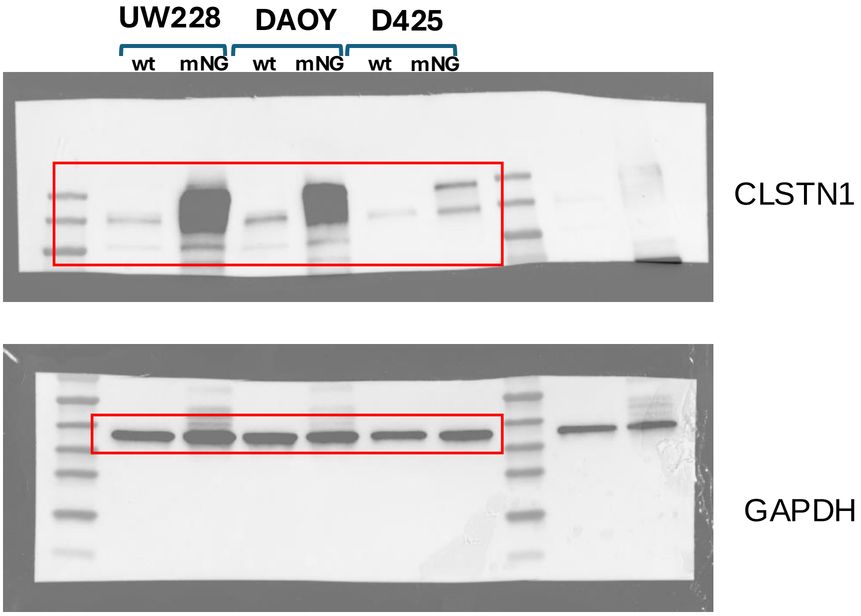

UW228
DAOY
D425
wt
mNG
wt
mNG
wt
mNG
CLSTN1
GAPDH

Fig. 5D

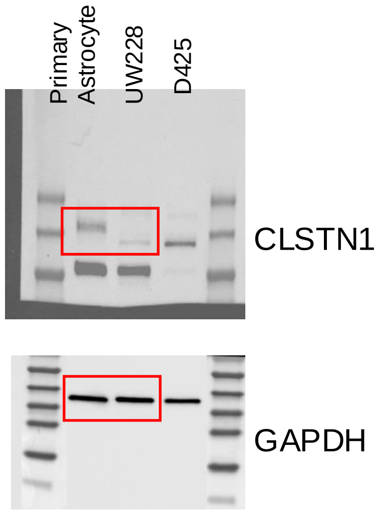
Primary Astrocyte
UW228
D425
CLSTN1
GAPDH

Fig. 6A

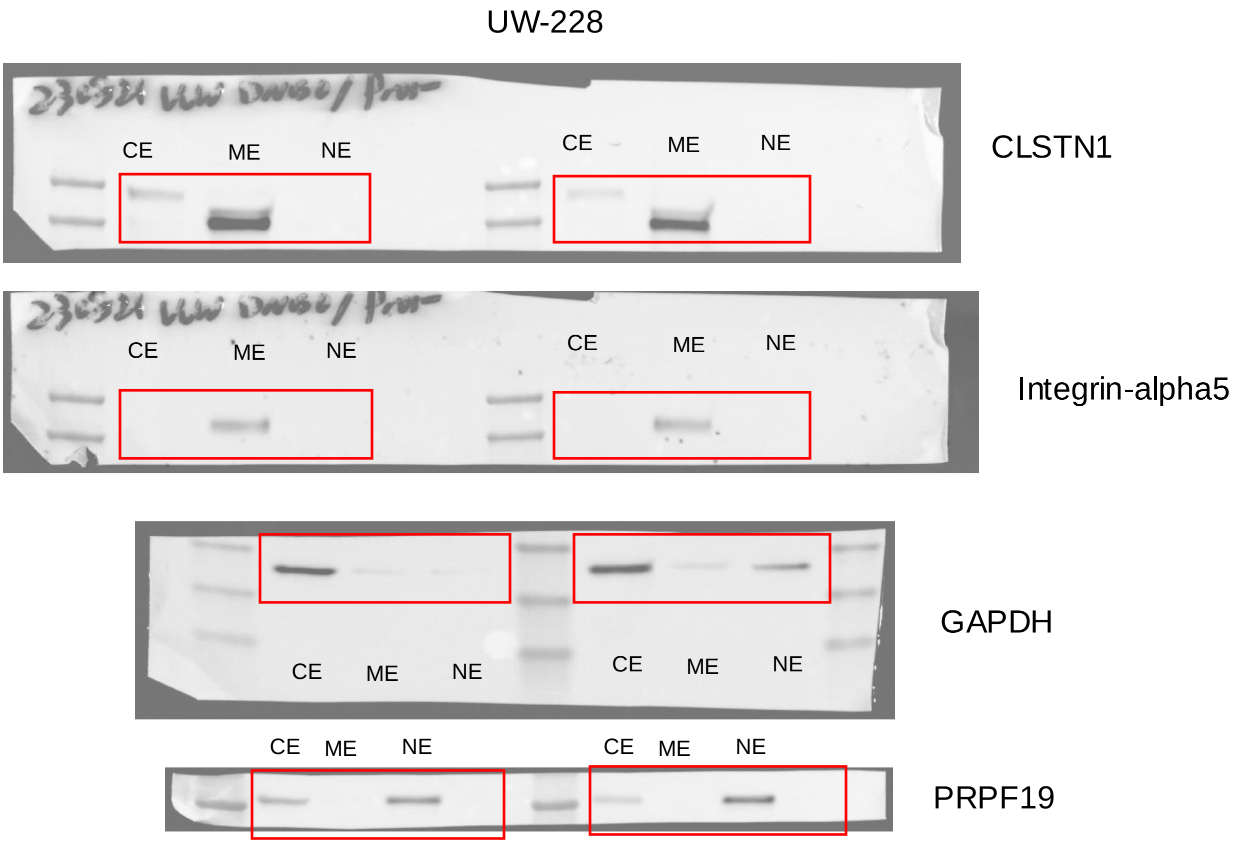
UW-228
CE
ME
NE
CE
ME
NE
CLSTN1
CE
ME
NE
CE
ME
NE
Integrin-alpha5
CE
ME
NE
CE
ME
NE
GAPDH
CE
ME
NE
CE
ME
NE
PRPF19

Fig. S3A

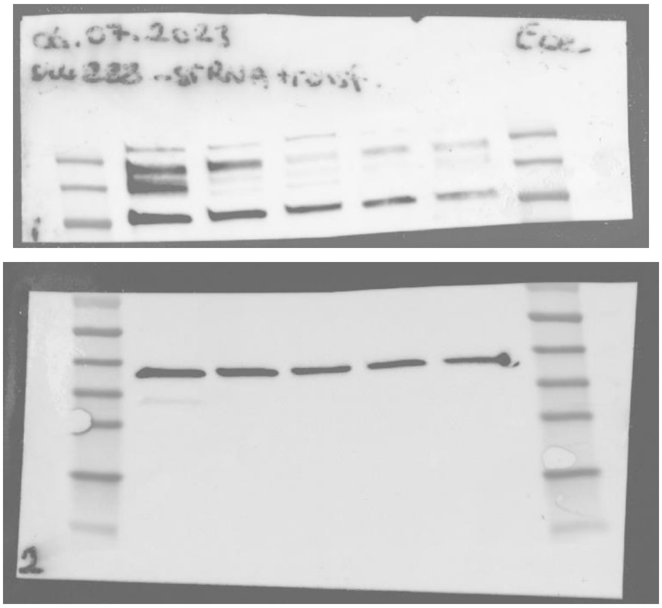

CLSTN1

GAPDH

Fig. S3B

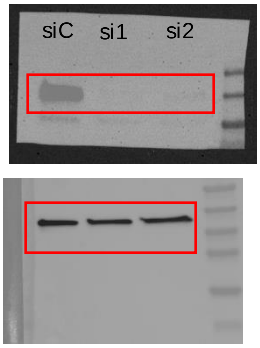

CLSTN1

GAPDH
