# Supplementary material for: MAP4 kinase-regulated reduced CLSTN1 expression in medulloblastoma is associated with increased invasiveness
Source: Sci Rep. 2025 Jan 6;15:946. doi: 10.1038/s41598-024-84753-x (PMC11704044; doi:10.1038/s41598-024-84753-x)
Supplement: Supplementary file 11 — Supplementary Material 11 [file 41598_2024_84753_MOESM11_ESM.pdf]

Supplementary methods file

Table 1: *Cell lines used*

Name	Origin	Authentication/testing	Medium
ONS-76	Michael Taylor lab, Sickkids Toronto, Canada	Single Nucleotide Polymorphism (SNP) typing, 02/2022) Regular LookOut® Mycoplasma PCR Detection kit (#MP0035, Sigma Aldrich)	RMPI-1640 medium (R0883, Sigma) supplemented with GlutaMax (#35050-038, Gibco), 10% FBS (S0615, Sigma) and 1% penicillin/streptomycin (#15140-122, Gibco)
HD-MB03	Till Milde, DKFZ Heidelberg, Germany	Single Nucleotide Polymorphism (SNP) typing, 02/2022) Regular LookOut® Mycoplasma PCR Detection kit (#MP0035, Sigma Aldrich)	RMPI-1640 medium (R0883, Sigma) supplemented with GlutaMax (#35050-038, Gibco), 10% FBS (S0615, Sigma) and 1% penicillin/streptomycin (#15140-122, Gibco)
UW228	John Silber, Seattle, USA	Single Nucleotide Polymorphism (SNP) typing, 02/2022) Regular LookOut® Mycoplasma PCR Detection kit (#MP0035, Sigma Aldrich)	DMEM medium (Gibco, 11965092) supplemented with GlutaMax (#35050-038, Gibco), 10% FBS (S0615, Sigma) and 1% penicillin/streptomycin (#15140-122, Gibco)
D425-Med (D425)	Henry Friedman lab, Duke University, UK	Single Nucleotide Polymorphism (SNP) typing, 02/2022) Regular LookOut® Mycoplasma PCR Detection kit (#MP0035, Sigma Aldrich)	IMEM medium (#10373-017, Gibco) supplemented with GlutaMax (#35050-038, Gibco), 10% FBS (S0615, Sigma) and 1% penicillin/streptomycin (#15140-122, Gibco)
D283-Med (D283)	ATTC	Single Nucleotide Polymorphism (SNP) typing, 02/2022) Regular LookOut® Mycoplasma PCR Detection kit (#MP0035, Sigma Aldrich)	IMEM medium (#10373-017, Gibco) supplemented with GlutaMax (#35050-038, Gibco), 10% FBS (S0615, Sigma) and 1% penicillin/streptomycin (#15140-122, Gibco)
iHNA (astrocytic cell line)	Ana Guerreiro Stücklin, University Children's Hospital Zürich	Regular LookOut® Mycoplasma PCR Detection kit (#MP0035, Sigma Aldrich)	DMEM medium (Gibco, 11965092) supplemented with GlutaMax (#35050-038, Gibco), 10% FBS (S0615, Sigma), 1% penicillin/streptomycin (#15140-122, Gibco) and 1% sodium pyruvate.
Primary murine cerebellar astrocytes	Prepared in house Procedure was approved by the cantonal authority under licence ZH079/2023_OCSC_2023	Regular LookOut® Mycoplasma PCR Detection kit (#MP0035, Sigma Aldrich)	DMEM medium (Gibco, 11965092) supplemented with GlutaMax (#35050-038, Gibco), 10% FBS (S0615, Sigma), 1% penicillin/streptomycin (#15140-122, Gibco) and 1% sodium pyruvate.

***Table 2**: Primary antibodies used in Western Blot*

Antibody	Source	Dilution	Company	Catalog Number
CLSTN1	Rabbit	1:1000	Abcam	134130
N-cadherin	Rabbit	1:1000	Cell Signaling Technology	13116T
Integrin-α-5	Rabbit	1:1000	Cell Signaling Technology	4705
GAPDH	Rabbit	1:1000	Cell Signaling Technology	2118L
Histone H3	Rabbit	1:1000	Cell Signaling Technology	44995
Vimentin	Rabbit	1:1000	Cell Signaling Technology	57415
C-Jun	Rabbit	1:1000	Cell Signaling Technology	9165

***Table 3:** Primary antibodies used in FACS*

Antibody	Source	Dilution	Company	Catalog Number
CLSTN1 (Biotin-conjugated)	Rabbit	1:100	ABIN	6876422
IgG Isotype Control (PE-conjugated)	Rabbit	1:300	Cell Signaling Technology	5742

***Table 4:** Secondary antibodies used in IFA*

Antibody	Source	Dilution	Company	Catalog Number
CLSTN1	Rabbit	1:100	Abcam	134130
Connexin 43 (Cx43)	Rabbit	1:1000	Millipore	C6219
Afadin 6 (AF-6)	Rabbit	1:1000	Millipore	C6219
N-Cadherin	Rabbit	1:1000	Thermo Fisher Scientific	PA5-83130
N-cadherin	Rabbit	1:400	Cell Signaling Technology	13116T
GFAP	Goat	1:250	Abacam	Ab53554

***Table 5:** Secondary Antibodies Used in Western Blot*

Antibody	Source	Dilution	Company	Catalog Number
Anti-rabbit IgG, HRP-linked	Goat	1:5000	Cell Signaling Technology	7074S
Anti-mouse IgG, HRP-linked	Horse	1:5000	Cell Signaling Technology	7076S

***Table 6**: Secondary antibodies used in FACS*

Antibody	Source	Dilution	Company	Catalog Number
PE Streptavidin	Rabbit	1:200	BioLegend	405203

***Table 7:** Secondary antibodies used in IFA*

Antibody	Source	Dilution	Company	Catalog Number
Alexa Fluor 647 – Anti-Rabbit IgG (H+L)	Donkey	1:250	Jackson ImmunoResearch	711-605-152
Alexa Fluor 421-Anti-Goat IgG (H+L)	Donkey	1:200	Jackson ImmunoResearch	705-675-147

Primary and secondary antibodies used in Western Blot, FACS, and IFA were diluted with 1X TBS-T 5% non-fat dry milk, 2% FBS in PBS, and 5% FBS in PBS, respectively.

Table 7: *siRNAs*

Target Gene	Company	Catalog Number
siCtrl (scrambled)	Dharmacon	D-001210-02-05
CLSTN1 (si1-CLSTN1)	Dharmacon	J-020393-05-0002
CLSTN1 (si2-CLSTN1)	Dharmacon	J-020393-06-0002
CLSTN1 (si3-CLSTN1)	Dharmacon	J-020393-07-0002
CLSTN1 (si4-CLSTN1)	Dharmacon	J-020393-08-0002
MAP4K4 (siMAP4K4)		

Table 8: *Lentiviral Vectors*

Vector Name	Vector ID	Company	Selection Marker	
pLV[Exp]-Bsd-CMV>hCLSTN1[NM_001009566.3](ns):3xGGGGS: mNeonGreen	*VB230510-1232gzf*	VectorBuilder	Bsd (blasticidin resistance gene)	
pLV[Exp]-Bsd-CMV>hCLSTN1[NM_001009566.3]/3xGGGGS	*VB230510-1225fgu* /V5.	VectorBuilder	Bsd (blasticidin resistance gene)	

CLSTN1-coding sequences in lentiviral vectors:

pLV_Bsd-CMV_hCLSTN1_3xGGGGS_V5:

MGGRRVRWEVYISRAGLVNRQIQVCTKKQAATMLRRPAPALAPAARLLLAGLLCGGGVWAARVNKHKPWLEPTYH
GIVTENDNTVLLDPPLIALDKDAPLRFAESFEVTVTKEGEICGFKIHGQNVPFDAVVVDKSTGEGVIRSKEKLDC
ELQKDYSFTIQAYDCGKGPDGTNVKKSHKATVHIQVNDVNEYAPVFKEKSYKATVIEGKQYDSILRVEAVDADCS
PQFSQICSYEIITPDVPFTVDKDGYIKNTEKLNYGKEHQYKLTVTAYDCGKKRATEDVLVKISIKPTCTPGWQGW
NNRIEYEPGTGALAVFPNIHLETCDEPVASVQATVELETSHIGKGCDRDTYSEKSLHRLCGAAAGTAELLPSPSG
SLNWTMGLPTDNGHDSDQVFEFNGTQAVRIPDGVVSVSPKEPFTISVWMRHGPFGRKKETILCSSDKTDMNRHHY
SLYVHGCRLIFLFRQDPSEEKKYRPAEFHWKLNQVCDEEWHHYVLNVEFPSVTLYVDGTSHEPFSVTEDYPLHPS
KIETQLVVGACWQEFSGVENDNETEPVTVASAGGDLHMTQFFRGNLAGLTLRSGKLADKKVIDCLYTCKEGLDLQ
VLEDSGRGVQIQAHPSQLVLTLEGEDLGELDKAMQHISYLNSRQFPTPGIRRLKITSTIKCFNEATCISVPPVDG
YVMVLQPEEPKISLSGVHHFARAASEFESSEGVFLFPELRIISTITREVEPEGDGAEDPTVQESLVSEEIVHDLD
TCEVTVEGEELNHEQESLEVDMARLQQKGIEVSSSELGMTFTGVDTMASYEEVLHLLRYRNWHARSLLDRKFKLI
CSELNGRYISNEFKVEVNVIHTANPMEHANHMAAQPQFVHPEHRSFVDLSGHNLANPHPFAVVPSTATVVIVVCV
SFLVFMIILGVFRIRAAHRRTMRDQDTGKENEMDWDDSALTITVNPMETYEDQHSSEEEEEEEEEEESEDGEEED
DITSAESESSEEEEGEQGDPQNATRQQQLEWDDSTLSYSGGGGSGGGGSGGGGSGKPIPNPLLGLDST

VB230510_pLV_Bsd_hCLSTN1_3xGGGGS_mNeonGreen

MGGRRVRWEVYISRAGLVNRQIQVCTKKQAATMLRRPAPALAPAARLLLAGLLCGGGVWAARVNKHKPWLEPTYH
GIVTENDNTVLLDPPLIALDKDAPLRFAESFEVTVTKEGEICGFKIHGQNVPFDAVVVDKSTGEGVIRSKEKLDC
ELQKDYSFTIQAYDCGKGPDGTNVKKSHKATVHIQVNDVNEYAPVFKEKSYKATVIEGKQYDSILRVEAVDADCS
PQFSQICSYEIITPDVPFTVDKDGYIKNTEKLNYGKEHQYKLTVTAYDCGKKRATEDVLVKISIKPTCTPGWQGW
NNRIEYEPGTGALAVFPNIHLETCDEPVASVQATVELETSHIGKGCDRDTYSEKSLHRLCGAAAGTAELLPSPSG
SLNWTMGLPTDNGHDSDQVFEFNGTQAVRIPDGVVSVSPKEPFTISVWMRHGPFGRKKETILCSSDKTDMNRHHY
SLYVHGCRLIFLFRQDPSEEKKYRPAEFHWKLNQVCDEEWHHYVLNVEFPSVTLYVDGTSHEPFSVTEDYPLHPS
KIETQLVVGACWQEFSGVENDNETEPVTVASAGGDLHMTQFFRGNLAGLTLRSGKLADKKVIDCLYTCKEGLDLQ
VLEDSGRGVQIQAHPSQLVLTLEGEDLGELDKAMQHISYLNSRQFPTPGIRRLKITSTIKCFNEATCISVPPVDG
YVMVLQPEEPKISLSGVHHFARAASEFESSEGVFLFPELRIISTITREVEPEGDGAEDPTVQESLVSEEIVHDLD
TCEVTVEGEELNHEQESLEVDMARLQQKGIEVSSSELGMTFTGVDTMASYEEVLHLLRYRNWHARSLLDRKFKLI
CSELNGRYISNEFKVEVNVIHTANPMEHANHMAAQPQFVHPEHRSFVDLSGHNLANPHPFAVVPSTATVVIVVCV

SFLVFMIILGVFRIRAAHRRTMRDQDTGKENEMDWDDSALTITVNPMETYEDQHSSEEEEEEEEEEESEDGEEED
DITSAESESSEEEEGEQGDPQNATRQQQLEWDDSTLSYSGGGGSGGGGSGGGGSMVSKGEEDNMASLPATHELHI
FGSINGVDFDMVGQGTGNPNDGYEELNLKSTKGDLQFSPWILVPHIGYGFHQYLPYPDGMSPFQAAMVDGSGYQV
HRTMQFEDGASLTVNYRYTYEGSHIKGEAQVKGTGFPADGPVMTNSLTAADWCRSKKTYPNDKTIIS
